# Impact of knowledge of the 2017 classification for periodontal and peri-implant diseases on diagnostic accuracy among dental students

**DOI:** 10.3389/fdmed.2025.1558380

**Published:** 2025-06-17

**Authors:** Shatha Bamashmous, Abdullaziz Farahat, Waleed Alharbi, Arwa Banjar, Amal Jamjoom, Arwa Badahdah

**Affiliations:** ^1^ Department of Periodontology, Faculty of Dentistry, King Abdulaziz University, Jeddah, Saudi Arabia; ^2^ Faculty of Dentistry, King Abdulaziz University, Jeddah, Saudi Arabia

**Keywords:** classification, dental students, diagnosis, knowledge, periodontitis

## Abstract

**Background:**

Periodontal diseases are a significant global health problem and have been associated with various systemic diseases. The American Academy of Periodontology (AAP) and the European Federation of Periodontology (EFP) introduced a new classification system for periodontal and peri-implant diseases in 2017. However, the complexity of this new classification has presented some challenges in its implementation compared to the 1999 classification system. This study evaluates the impact of dental students’ knowledge of the 2017 classification on their ability to diagnose periodontal diseases accurately.

**Materials and methods:**

This cross-sectional study enrolled 146 fifth- and sixth-year dental students from King Abdulaziz University in Jeddah, Saudi Arabia. A self-reported questionnaire was employed to assess students’ knowledge of the 2017 periodontal classification system and their diagnostic abilities across five clinical cases. Students were classified into low, moderate, and high knowledge groups based on their knowledge questionnaire scores. Statistical analyses assessed the relationship between classification knowledge and diagnostic accuracy and compared these measures across different groups.

**Results:**

In the study, 41.10% of participants achieved high knowledge scores (14–16 correct answers), while 32.88% and 26.03% were categorized into moderate and low knowledge groups, respectively. There was no significant association between knowledge levels and diagnostic accuracy, except for one specific clinical case. While sixth-year students scored significantly higher in the knowledge assessment than fifth-year students (*p* = 0.005), their diagnostic accuracy did not significantly differ. The main challenge, as reported by students, was the discrepancy between case complexity and their clinical experience (28.1%).

**Conclusion:**

This study found no significant impact of dental students’ knowledge of the 2017 periodontal classification system on diagnostic accuracy. Challenges such as discrepancies between case complexity and clinical experience and teaching inconsistencies underscore the need for enhanced clinical simulations, case-based learning, and targeted educational training to improve diagnostic accuracy and clinical competence.

## Introduction

Periodontal diseases present a substantial global health challenge, affecting nearly half the global population, and are linked to various systemic diseases and conditions (1, 2). The establishment of an accurate diagnosis and the development of a comprehensive treatment plan is essential for the prevention of disease progression and the achievement of optimal clinical outcomes. In 2017, the American Academy of Periodontology (AAP) and the European Federation of Periodontology (EFP) introduced a new classification system that was in accordance with the most recent evidence (3). It implements staging and grading to improve diagnostic consistency and accuracy in periodontitis cases. The Severity and complexity of the case define the stage, while disease progression and risk factors including smoking and diabetes define the grade of a periodontitis case (4). Staging might give the clinician an initial conception of the treatment plan and case management, while the grading provides insight into the response to therapy and the future risk of recurrence (5). Successfully implementing the new system requires a thorough assessment of various findings to distinguish between disease stages and correctly assign a grade (6, 7). However, the increased complexity of this system compared to the 1999 classification has resulted in implementation challenges, particularly in educational and clinical settings (8, 9).

Since the introduction of the 2017 periodontal classification, several studies have examined its implementation and evaluated diagnostic agreement among both clinicians and students (9–16). Within educational settings, dental students have demonstrated low diagnostic accuracy, underscoring the challenges they face in understanding and applying the new classification system (15, 16). Several factors influence students’ diagnostic performance and confidence, including integrating theoretical knowledge into clinical practice and the consistency of instructional methods (17). Successfully addressing these challenges is essential for optimizing patient outcomes and providing clinicians with the knowledge and skills needed to effectively manage the global burden of periodontal diseases. Studies have demonstrated that faculty calibration establishes standardized evaluation criteria and teaching methods, while case-based learning helps students connect theoretical knowledge with clinical practice, ultimately improving diagnostic proficiency (18–20). This highlights the need for further research to validate and enhance the reliability of the new classification, ensuring its applicability across diverse clinical settings. Accordingly, this study evaluated the accuracy of fifth- and sixth-year dental students’ diagnoses and investigated the impact of their knowledge of the 2017 periodontal classification on their diagnostic performance.

## Materials and methods

### Study design and population

The Research Ethics Committee at the Faculty of Dentistry, King Abdulaziz University reviewed and approved this cross-sectional study (Proposal no. 200-11-23). The minimum required sample size of 128 participants was determined through power calculations conducted using G*Power 3.1.9.4 with an effect size of 0.25, an alpha level of 0.05, and a power of 0.80. The study population included fifth- and sixth-year undergraduate dental students from the Faculty of Dentistry at King Abdulaziz University (KAU) during the 2023/2024 academic year. All eligible fifth- and sixth-year students, a total of 216, were invited to participate. Of these, 146 students consented, forming the final study sample, with an overall response rate of 67.6%. No exclusion criteria were applied in the selection of the participant cohort. Dental students complete a six-year program at KAU, with periodontics integrated into the final three years via didactic coursework and clinical practice. As part of their periodontal training, students in both groups received formal instruction on the 2017 periodontal classification system through lectures, case-based learning, and supervised clinical application. All participants provided informed consent, and participation was voluntary. Throughout the study, anonymity and confidentiality were assured.

### Questionnaire design and administration

A self-administered questionnaire was developed to assess participants’ understanding of the 2017 periodontal classification system. It consisted of three sections: the first included nine questions evaluating students’ self-assessed confidence in diagnosing periodontal diseases and their attitudes toward the 2017 classification system using a five-point scale. The second section comprised sixteen multiple-choice questions covering key aspects of staging, grading, and diagnostic criteria. The final section involved the diagnosis of five clinical cases, each accompanied by detailed clinical information, including medical history such as smoking and diabetes, dental history, full periodontal charts, and full mouth radiographs. The periodontal charts included probing depths (PD), clinical attachment loss (CAL), furcation, mobility, missing teeth, plaque index, and bleeding on probing. Clinical cases were selected to include a range of diagnostic complexities, covering different stages and grades of periodontitis according to the 2017 classification system (3, 4). These cases were chosen to assess students’ ability to apply theoretical knowledge to clinical decision-making. The invitation link was sent to students via their university email accounts, and the questionnaire was administered online using Google Forms (Google LLC, Mountain View, California, USA) between December 2023 and April 2024. Students completed the questionnaire remotely at their convenience without direct supervision. Two experienced periodontists validated all three sections of the questionnaire for content relevance and determined the appropriate diagnosis for the five cases in accordance with the 2017 periodontal classification guidelines (3, 4). The clarity of the questionnaire was confirmed through a pilot test involving 16 students, who were excluded from the final study sample; the results indicated no need for significant modifications.

### Participant grouping and diagnostic scoring

Based on their knowledge scores from the second section of the questionnaire, participants were categorized into low (0–11 correct answers), moderate (12, 13), and high (14–16) knowledge groups. The total diagnostic accuracy score for each student was determined by summing the scores from the five clinical cases, with each case contributing up to 3 points based on the correctness of the diagnosis, staging, and grading, resulting in a maximum overall score of 15. The average total diagnostic accuracy scores were then calculated for each knowledge group.

### Statistical analysis

The diagnostic accuracy for each of the five clinical cases was assessed by evaluating the agreement between student diagnoses and reference diagnoses established by two experienced periodontists following the 2017 periodontal classification. Descriptive statistics summarize nominal and categorical variables as frequencies and percentages and continuous variables are reported as means and standard deviations. Mann–Whitney U and chi-square tests were used to compare attitudes and confidence between fifth- and sixth-year students, while chi-square and independent *t*-tests were performed to determine differences in knowledge and diagnostic accuracy. Fisher's Exact Test was conducted to compare the challenges faced by 5th-year and 6th-year students. A one-way ANOVA was used to assess differences in total diagnostic accuracy scores across groups, followed by *post hoc* tests to identify specific group differences. A significance threshold of *p* < 0.05 was applied to all analyses.

## Results

### Students’ attitude, confidence, and challenges in using the 2017 periodontal classification

**Figure 1:**
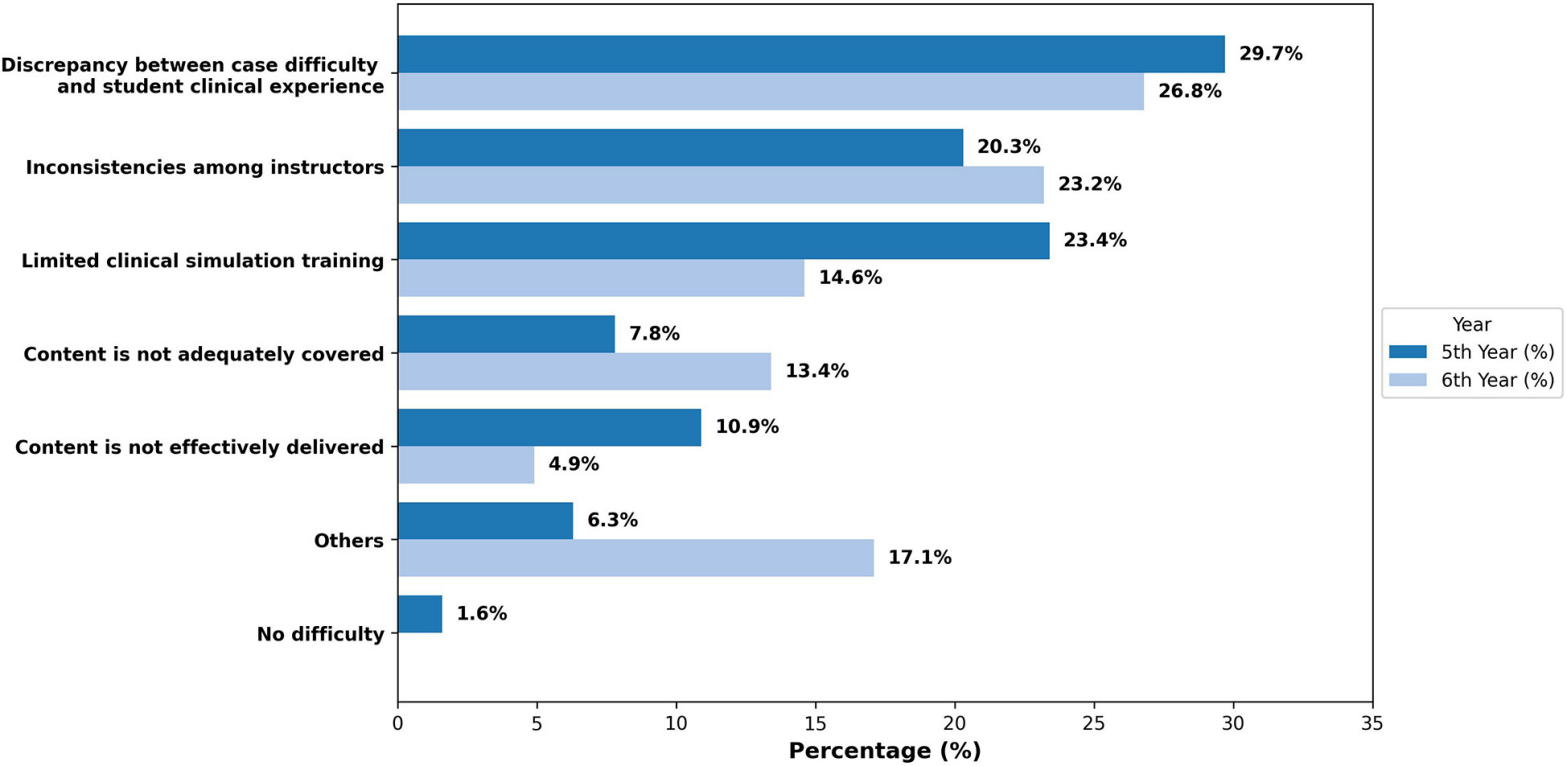
Challenges faced by students with the 2017 periodontal classification. The bars represent the proportion of 5th- and 6th-year dental students who reported difficulties in applying the 2017 Periodontal Classification system. The discrepancy between case difficulty and student clinical experience was the most frequently reported challenge in both groups (28.1%).

A total of 146 dental students participated, with 64 fifth-year students (43.8%) and 82 sixth-year students (56.2%), Table 1. The gender distribution was almost equal, and the overall GPA of the participants was considered high, averaging 4.3 ± 0.4 out of 5. The self-assessment results for knowledge and confidence in periodontal diagnosis are shown in Table 2. Results showed students rated their knowledge and confidence in periodontal diagnosis similarly at an average of 3.8 ± 0.8, with no significant difference between groups. Both groups agreed that periodontal diagnosis significantly impacts patient care and treatment planning (4.1 ± 1.0). Sixth-year students reported diagnosing more cases of periodontitis (5.7 ± 3.7) than fifth-year students (4.5 ± 5.2), *p* < 0.001. Overall, 86.3% of students believed their education adequately prepared them for periodontal diagnosis. Students reported several difficulties regarding the new classification implementation, Figure 1. The most common challenges included a discrepancy between case complexity and clinical experience (28.1%) and inconsistencies among instructors (21.9%). Limited clinical simulation training was reported by 18.5% of students, with more fifth-year (23.4%) than sixth-year (14.6%) students, Supplementary Table 1.

**Table 1:** Characteristics of study participants.

Variables [Mean ± SD or *n* (%)]		Total (*N* = 146)	5th year (*n* = 64)	6th year (*n* = 82)	*p*-value
Age	Mean ± SD	23.6 ± 1.1	23.2 ± 1.2	23.9 ± 1.0	<0.001‡*
Gender	Male	74 (50.7)	32 (50.0)	42 (51.2)	0.884†
	Female	72 (49.3)	32 (50.0)	40 (48.8)	
GPA	Mean ± SD	4.3 ± 0.4	4.4 ± 0.4	4.3 ± 0.4	0.301‡

Chi-square (†) and independent sample *t*-tests (‡) were used to assess differences between groups. Statistically significant differences are denoted by *P* < 0.05 (*).

**Table 2:** Self-assessment of attitudes and confidence regarding the 2017 classification system.

Questions [Mean ± SD or *n* (%)]	Total (*N* = 146)	5th Year (*n* = 64)	6th Year (*n* = 82)	*p*-value
How would you rate your knowledge of periodontal diagnosis? (1 = very limited, 5 = very extensive)	3.8 ± 0.8	3.9 ± 1.8	3.8 ± 1.7	0.351‡
To what extent do you believe that a solid understanding of periodontal diagnosis is important for a dental professional? (1 = not important at all, 5 = very important)	4.3 ± 1.0	4.4 ± 1.1	4.2 ± 0.9	0.008‡*
How confident are you in your ability to perform periodontal diagnosis effectively in a clinical setting? (1 = Not confident at all, 5 = Very confident)	3.8 ± 0.8	3.8 ± 0.9	3.9 ± 0.7	0.690‡
How interested are you in periodontal diagnosis as a subject of study? (1 = not interested at all, 5 = very interested)	3.0 ± 1.2	3.3 ± 1.2	3.3 ± 1.2	0.790‡
To what degree do you think periodontal diagnosis impacts overall patient care and treatment planning? (1 = no impact, 5 = significant impact)	4.1 ± 1.0	4.2 ± 1.1	4.1 ± 1.0	0.252‡
How do you view the role of periodontal diagnosis in your future dental practice? (1 = not important at all, 5 = very important)	4.0 ± 1.0	4.0 ± 1.2	4.0 ± 0.9	0.326‡
How many periodontitis cases have you diagnosed?	5.2 ± 4.4	4.5 ± 5.2	5.7 ± 3.7	<0.001‡*
Dental education adequately prepares students for periodontal diagnosis.	126 (86.3%)	54 (84.4%)	72 (87.8%)	0.550†

Chi-square (†) and Mann–Whitney *U* (‡) tests were used to assess differences between groups. Statistically significant differences are denoted by *p* < 0.05 (*).

### Students’ knowledge about diagnosis, staging, and grading of periodontitis

**Figure 2:**
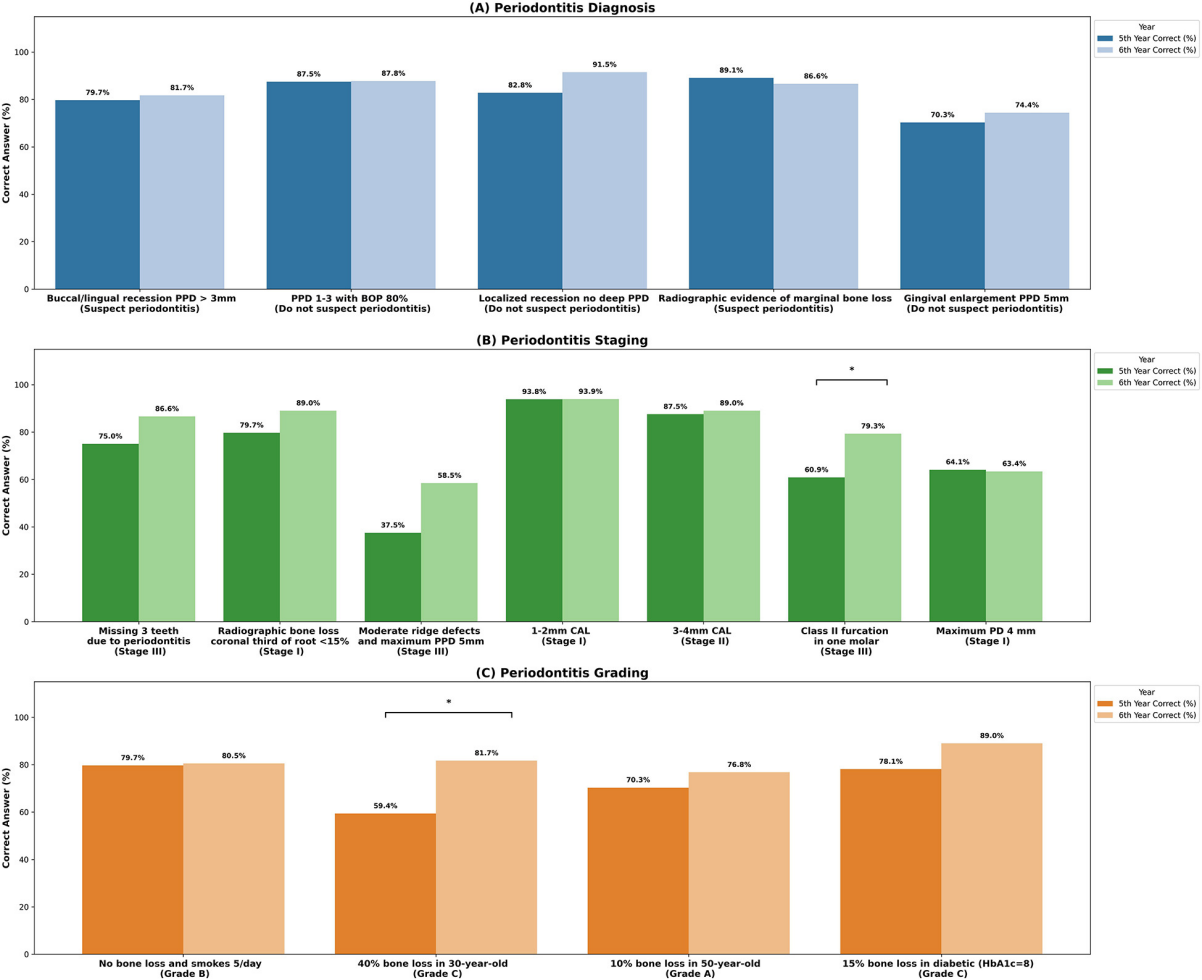
Students’ knowledge about diagnosis, staging, and grading of periodontitis. The bars represent the proportion of 5th- and 6th-year dental students who correctly diagnosed, staged, and graded periodontal diseases according to diagnostic criteria. **(A)** There was no significant difference between fifth- and sixth-year students in periodontitis diagnosis. However, **(B)** significant differences were found in staging Class II furcation as stage III (*p* = 0.046), and **(C)** grading 40% bone loss in a 30-year-old as Grade C (*p* = 0.007). Statistically significant differences are denoted by *p* < 0.05 (*).

The analysis of student responses revealed that most students correctly suspected periodontitis for buccal or lingual recession with PPD > 3 mm (80%) and radiographic bone loss (87.7%), Figure 2A. However, 27.4% incorrectly suspected periodontitis for gingival enlargement in maxillary anterior teeth with a PPD of 5 mm. For periodontitis staging based on signs and symptoms, 81.5% correctly classified missing three teeth due to periodontitis as Stage III. Only 49.3% correctly classified moderate ridge defects and a probing depth of 5 mm as Stage III. Additionally, significantly more 6th-year students (79.3%) than 5th-year students (64.1%) correctly classified Class II furcation as Stage III (*p* = 0.046), Figure 2B. For grading, most students (84.2%) correctly classified 15% bone loss in a diabetic patient (HbA1c = 8) as Grade C, with no significant differences between groups. For 40% bone loss in a 30-year-old, 71.9% correctly classified it as Grade C, with significantly more 6th-year students (81.7%) than 5th-year students (59.4%) (*p* = 0.007), Figure 2C. Overall, 6th-year students scored higher on knowledge questions (13.1 ± 2.5) than 5th-year students (11.9 ± 2.3) (*p* = 0.005), Supplementary Table 2.

### Students’ diagnosis accuracy across clinical cases

**Figure 3:**
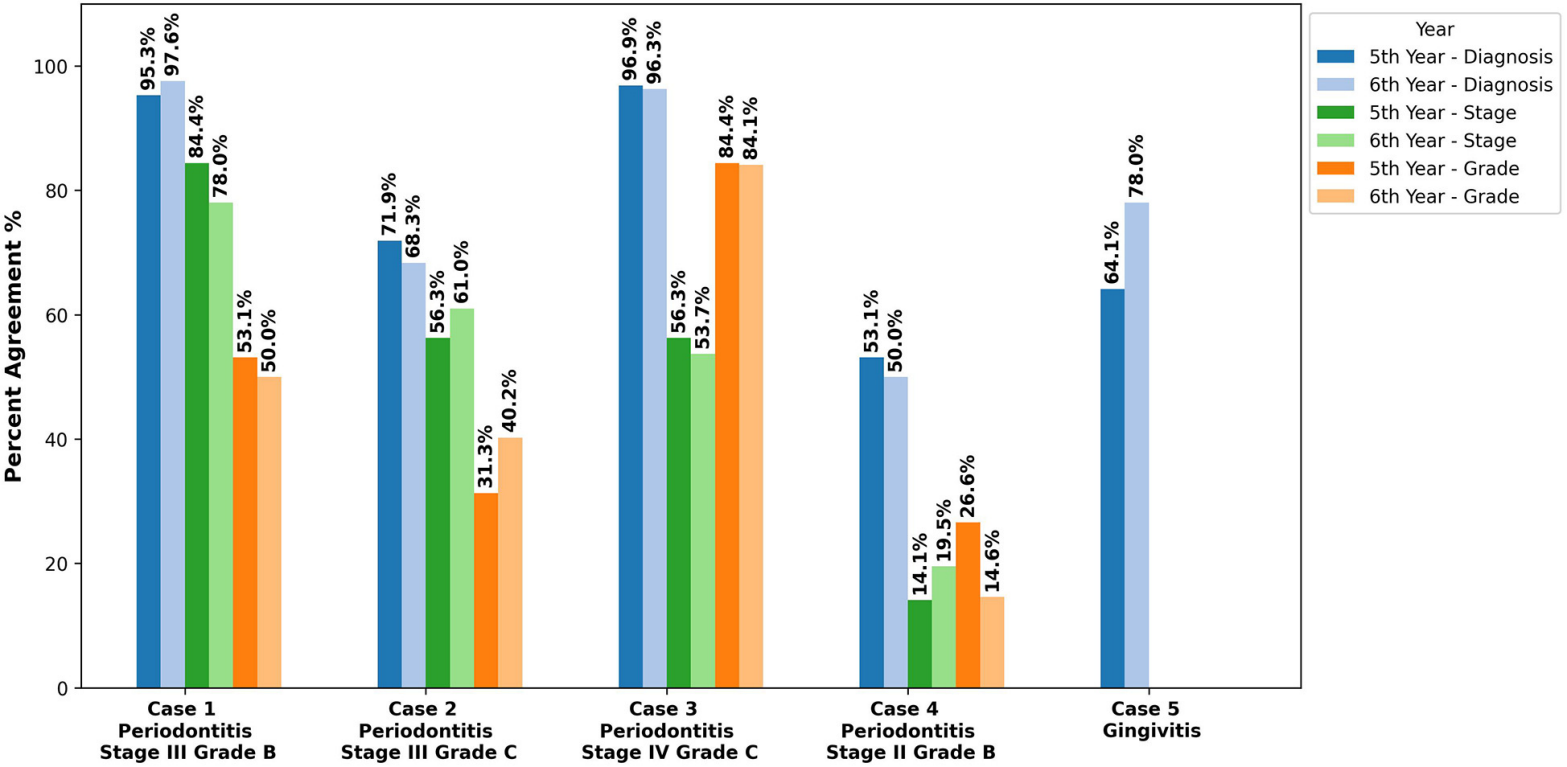
Percent agreement for diagnosis, staging, and grading across clinical cases. The bars represent the proportion of 5th- and 6th-year dental students who accurately determined the diagnosis, staging, and grading across five clinical cases. Case 1 had the highest accuracy, with 96% correctly diagnosing periodontitis, 80.8% identifying Stage III, and 51.4% assigning Grade B. In contrast, Case 4 had the lowest accuracy, with 51.4% correctly diagnosing periodontitis, only 17.1% identifying Stage II, and 19.9% assigning Grade B.

The accuracy of periodontal diagnosis varied across cases, as shown in Figure 3. Case 1 showed the highest accuracy, with 96% correctly diagnosing periodontitis, 80.8% identifying the correct stage (stage III), and 51.4% grading it as grade B, primarily justified by bone loss (41.8%). In contrast, case 4 had the lowest accuracy, with 51.4% correctly diagnosing periodontitis, only 17.1% identifying it as stage II, and 19.9% assigning Grade B. For Case 2, 69.9% diagnosed periodontitis, 58.9% correctly staged it as stage III, and 36.3% graded it as grade C, with percentage bone loss more frequently chosen by 5th-year students (53.1%) than 6th-year students (37.8%). Case 3 had the highest grading accuracy, with 84.2% correctly identifying grade C. In Case 5, 71.6% diagnosed it as gingivitis, while 23.3% incorrectly identified it as periodontitis. Across clinical cases, the bone loss percentage was the most frequently chosen justification for periodontitis diagnosis, with the highest percentage observed in Case 1 (41.8%) and Case 2 (44.5%). The average total diagnostic accuracy score was 9.3 ± 2.4 out of 15, with no significant difference between 5th-year (9.1 ± 2.6) and 6th-year students (9.5 ± 2.2) (*p* = 0.313), Supplementary Table 3.

### Association between students’ knowledge scores and diagnostic accuracy across periodontitis cases

Students were categorized into three knowledge groups: 0–11 (*n* = 38), 12–13 (*n* = 48), and 14–16 (*n* = 60), with average total diagnostic accuracy score increasing significantly as knowledge improved (*p* < 0.001), Table 3. In Case 1, staging accuracy was 84.2%, 87.5%, and 73.3% across the groups, respectively, with no significant differences. For Case 2, accuracy improved with higher knowledge scores (47.4% to 66.7%), though differences were not statistically significant (*p* = 0.147). In Case 3, the 14–16 group (66.7%) significantly outperformed the 0–11 group (39.5%) (*p* = 0.028). Staging accuracy in Case 4 was low across all groups (13.2% to 23.3%), while Case 5 showed relatively high accuracy across groups, with no significant differences observed (*p* = 0.545).

**Table 3:** Relationship between students’ knowledge scores and diagnostic accuracy.

Knowledge score (*n*)	Total diagnostic accuracy score (mean ± SD)	Case 1 staging accuracy [*n* (%)]	Case 2 staging accuracy [*n* (%)]	Case 3 staging accuracy [*n* (%)]	Case 4 staging accuracy [*n* (%)]	Case 5 staging accuracy [*n* (%)]
0–11 (*n* = 38)	8.2 ± 2.8^a^	32 (84.2%)	18 (47.4%)	15 (39.5%)	5 (13.2%)	26 (68.4%)
12–13 (*n* = 48)	9.1 ± 2.4^a^	42 (87.5%)	28 (58.3%)	25 (52.1%)	6 (12.5%)	36 (75.0%)
14–16 (*n* = 60)	10.3 ± 1.7^b^	44 (73.3%)	40 (66.7%)	40 (66.7%)	14 (23.3%)	47 (78.3%)
*p*-value	< 0.001‡*	0.147	0.166	0.028†*	0.25	0.545

This table examines the association between students’ knowledge scores and their diagnostic accuracy score across five cases, as well as their accuracy in staging individual cases. Chi-square test (†) and ANOVA (‡) were used to assess differences between groups, with significance at *P* < 0.05 (*). Different letters indicate statistically significant differences in *post hoc* analysis.

## Discussion

While the new classification has been broadly accepted for its evidence-based approach, the study findings revealed some challenges dental students may encounter when applying this system in clinical practice. This study aimed to assess students’ knowledge level regarding the new classification system and correlate these levels with their ability to diagnose real clinical cases. Our results suggest that understanding the classification system is essential, but it does not necessarily lead to improved diagnostic accuracy, especially in complex clinical cases. Sixth-year students scored significantly higher on knowledge assessments (13.1 ± 2.5) compared to fifth-year students (11.9 ± 2.3) (*p* = 0.005); however, their diagnostic accuracy across clinical cases did not show a significant difference as sixth-year students had an average total diagnostic accuracy score of 9.5 ± 2.2, while fifth-year students scored 9.1 ± 2.6 (*p* = 0.313). Fifth- and sixth-year students were selected for this study because they had recently completed formal instruction on the 2017 classification system and were expected to demonstrate competency in periodontal diagnosis before graduation. Despite higher theoretical knowledge, sixth-year students showed no significant improvement in diagnostic accuracy, likely due to limited clinical exposure and reliance on structured assessments. Similarly, Gandhi et al. reported diagnostic accuracy ranged from 22.6% to 27.6% by dental students from three different dental schools, underscoring the difficulty of translating theoretical knowledge into clinical practice (15).

The analysis of student responses revealed significant variability in diagnosing and staging periodontitis, with performance varying based on case complexity. Students performed well on cases with evident periodontal destruction, such as Case 1 (Periodontitis stage III), where 96.6% identified the correct diagnosis and 80.8% accurately staged the case. However, cases with minimal periodontal involvement, like Case 4 (Periodontitis stage II), posed significant challenges, with only 51.4% identifying the correct diagnosis and 17.1% staging it accurately. The lower diagnostic accuracy in Case 4 could be due to its borderline presentation, with minimal bone and attachment loss, making it difficult for students to distinguish early-stage periodontitis from gingivitis, leading to an underestimation of periodontal disease. Abrahamian et al. reported inter-examiner reliability challenges in distinguishing between periodontitis stages, particularly in cases requiring subjective assessments, with an agreement rate of 68.7% for staging (6). The low percentage of bone loss in stage I and II cases often led students to misclassify them as gingivitis, while distinguishing between stages III and IV proved to be challenging due to their similar levels of attachment and bone loss, requiring evaluation of tooth loss history or complexity criteria with subjective interpretations (14). Additionally, cases involving hopeless teeth or those nearing extraction further complicate staging, as determining tooth prognosis remains highly subjective (6).

Our findings revealed that increased knowledge does not correlate with improved diagnostic performance, with no significant differences observed among students with low, medium, or high knowledge scores, except for Case 3 (Periodontitis stage IV), where staging accuracy was significantly higher among students scoring 14–16 (66.7%) compared to those scoring 0–11 (39.5%) *(p* *=* 0.028). This may indicate that extremely severe cases are more easily identifiable by students with greater knowledge. Additionally, this study demonstrated higher grading accuracy than staging, likely due to the clear and specific criteria outlined in the 2017 classification system. Similarly, Ravidà et al. reported higher inter-examiner agreement for grading (82%) compared to staging (76.6%) (11). These findings highlight that consistent application of staging and grading criteria remains challenging, particularly in complex cases, as “gray zones” in the classification criteria contribute to significant variability, reducing rater consistency and posing challenges for students and clinicians in applying the new classification system (6).

While 86.3% of students in this study agreed that their dental education prepared them for periodontal diagnosis, several challenges in applying the 2017 Periodontal Classification System were reported. A discrepancy between case difficulty and student clinical experience was one of the main challenges, affecting 28.1% of students. Inadequate exposure to complex cases contributed to diagnostic variability, emphasizing the need for comprehensive clinical simulations and structured case-based training in periodontal education (11). Advanced training and clinical exposure have been shown to enhance diagnostic accuracy, as postgraduate periodontology students achieved a higher degree of diagnosis agreement (Kappa = 0.55) than undergraduates (12).

Additionally, students reported challenges with inconsistencies in instructor feedback and assessment, which may have contributed to diagnostic variability. Regular calibration programs and standardized student assessments remain crucial for improving diagnostic accuracy and minimizing student variability (13). Faculty calibration sessions demonstrated to significantly enhance diagnostic consistency, resulting in improved agreement on periodontal disease classification and treatment planning (20). Similarly, structured consensus training can help minimize variations in diagnosis, improve student performance, and reduce variability in treatment decisions (18, 19).

Students reported that insufficient content coverage (11.0%) and ineffective content delivery (7.5%) may have impacted their ability to apply the 2017 Periodontal Classification System, negatively affecting learning and contributing to lower performance in periodontal diagnosis (17). Studies in health education have shown that blended learning, which integrates conventional in-person instruction with digital and interactive components, leads to superior knowledge retention and comprehension compared to traditional learning approaches (21). Huynh et al. found that implementing an online module with interactive case quizzes significantly enhanced students’ understanding of the 2017 Periodontal Classification System, with over 80% of participants favoring it over traditional lectures (22).

This study was conducted in a single institutional setting, which may limit the generalizability of the results due to institution-specific curricular structures and clinical training approaches. Moreover, self-reported questionnaires may introduce potential bias, as the self-administered and remotely completed format prevented verification of whether students utilized external resources, potentially influencing their responses. Future research with multi-institutional studies, longitudinal designs, and larger sample sizes is recommended to validate these findings and assess the long-term impact of targeted educational interventions.

## Conclusion

This study found no significant impact of dental students’ knowledge of the 2017 periodontal classification system on their diagnostic accuracy. Challenges such as discrepancies between case complexity and clinical experience and teaching inconsistencies underscore the need for enhanced clinical simulations, case-based learning, and targeted educational training to improve dental students’ diagnostic accuracy and clinical competence.

## Data Availability

The original contributions presented in the study are included in the article/Supplementary Material, further inquiries can be directed to the corresponding author.
