# Supplementary material for: Impact of knowledge of the 2017 classification for periodontal and peri-implant diseases on diagnostic accuracy among dental students
Source: Front Dent Med. 2025 Jun 17;6:1558380. doi: 10.3389/fdmed.2025.1558380 (PMC12209212; doi:10.3389/fdmed.2025.1558380)
Supplement: Supplementary file 1 [file Table1.docx]

**Supplemental Table 1: Challenges Faced by Students with the 2017 Periodontal Classification.**

| **Challenges** | **Total**  **(N=146)** | **5th Year (n=64)** | **6th Year (n=82)** | **p-value** |
| --- | --- | --- | --- | --- |
| **Discrepancy between case difficulty and student clinical experience** | 41 (28.1%) | 19 (29.7%) | 22 (26.8%) | 0.715 |
| **Inconsistencies among instructors** | 32 (21.9%) | 13 (20.3%) | 19 (23.2%) | 0.84 |
| **Limited clinical simulation training** | 27 (18.5%) | 15 (23.4%) | 12 (14.6%) | 0.201 |
| **Content is not adequately covered** | 16 (11.0%) | 5 (7.8%) | 11 (13.4%) | 0.424 |
| **Content is not effectively delivered** | 11 (7.5%) | 7 (10.9%) | 4 (4.9%) | 0.212 |
| **Other difficulties** | 18 (12.3%) | 4 (6.3%) | 14 (17.1%) | 0.074 |
| **No difficulty** | 1 (0.7%) | 1 (1.6%) | 0 (0%) | 0.438 |

The responses were categorized into predefined challenges, with percentages indicating the proportion of 5th- and 6th-year dental students reporting each difficulty. Fisher's Exact test (†) was used to assess differences between groups. Statistically significant differences are denoted by *p* < 0.05 (*).

**Supplemental Table 2: Comparison of Periodontal Diagnosis Knowledge, Staging, and Grading Among 5th and 6th Year Dental Students**

| **Questions** | **Signs and Symptoms** | **Responses** | **Total (N=146)** | **5th Year (n=64)** | **6th Year (n=82)** | **p-value** |
| --- | --- | --- | --- | --- | --- | --- |
| **Suspecting Periodontitis** | **Buccal or lingual recession with PPD > 3 mm** | Suspect periodontitis | 118 (80.8%) | 51 (79.7%) | 67 (81.7%) | 0.758 † |
|  |  | Do not suspect periodontitis | 28 (19.2%) | 13 (20.3%) | 15 (18.3%) |  |
|  | **PPD 1-3 with BOP 80%** | Suspect periodontitis | 18 (12.3%) | 8 (12.5%) | 10 (12.2%) | 0.956 † |
|  |  | Do not suspect periodontitis | 128 (87.7%) | 56 (87.5%) | 72 (87.8%) |  |
|  | **Localized recession with no deep probing depth** | Suspect periodontitis | 18 (12.3%) | 11 (17.2%) | 7 (8.5%) | 0.115 † |
|  |  | Do not suspect periodontitis | 128 (87.7%) | 53 (82.8%) | 75 (91.5%) |  |
|  | **Radiographic evidence of marginal bone loss** | Suspect periodontitis | 128 (87.7%) | 57 (89.1%) | 71 (86.6%) | 0.651 † |
|  |  | Do not suspect periodontitis | 18 (12.3%) | 7 (10.9%) | 11 (13.4%) |  |
|  | **Gingival enlargement in maxillary anterior with PPD of 5 mm** | Suspect periodontitis | 40 (27.4%) | 19 (29.7%) | 21 (25.6%) | 0.584 † |
|  |  | Do not suspect periodontitis | 106 (72.6%) | 45 (70.3%) | 61 (74.4%) |  |
| **Periodontitis Staging Based on Signs and Symptoms** | **Missing 3 teeth due to periodontitis** | Stage I | 3 (2.1%) | 1 (1.6%) | 2 (2.4%) | 0.181 † |
|  |  | Stage II | 16 (11.0%) | 9 (14.1%) | 7 (8.5%) |  |
|  |  | Stage III | 119 (81.5%) | 48 (75.0%) | 71 (86.6%) |  |
|  |  | Stage IV | 8 (5.5%) | 6 (9.4%) | 2 (2.4%) |  |
|  | **Radiographic bone loss in coronal third of the root <15%** | Stage I | 124 (84.9%) | 51 (79.7%) | 73 (89.0%) | 0.233 † |
|  |  | Stage II | 17 (11.6%) | 10 (15.6%) | 7 (8.5%) |  |
|  |  | Stage III | 4 (2.7%) | 3 (4.7%) | 1 (1.2%) |  |
|  |  | Stage IV | 1 (0.7%) | 0 (0%) | 1 (1.2%) |  |
|  | **Moderate ridge defects and maximum probing depth is 5 mm** | Stage I | 8 (5.5%) | 5 (7.8%) | 3 (3.7%) | 0.084 † |
|  |  | Stage II | 62 (42.5%) | 33 (51.6%) | 29 (35.4%) |  |
|  |  | Stage III | 72 (49.3%) | 24 (37.5%) | 48 (58.5%) |  |
|  |  | Stage IV | 4 (2.7%) | 2 (3.1%) | 2 (2.4%) |  |
|  | **1-2 mm CAL** | Stage I | 137 (93.8%) | 60 (93.8%) | 77 (93.9%) | 0.494 † |
|  |  | Stage II | 8 (5.5%) | 3 (4.7%) | 5 (6.1%) |  |
|  |  | Stage III | 1 (0.7%) | 1 (1.6%) | 0 (0%) |  |
|  |  | Stage IV | 0 (0%) | 0 (0%) | 0 (0%) |  |
|  | **3-4 mm CAL** | Stage I | 1 (0.7%) | 0 (0%) | 1 (1.2%) | 0.548 † |
|  |  | Stage II | 129 (88.4%) | 56 (87.5%) | 73 (89.0%) |  |
|  |  | Stage III | 15 (10.3%) | 8 (12.5%) | 7 (8.5%) |  |
|  |  | Stage IV | 1 (0.7%) | 0 (0%) | 1 (1.2%) |  |
|  | **Class II furcation in one molar** | Stage I | 6 (4.1%) | 5 (7.8%) | 1 (1.2%) | 0.046 †* |
|  |  | Stage II | 32 (21.9%) | 16 (25.0%) | 16 (19.5%) |  |
|  |  | Stage III | 106 (72.6%) | 41 (64.1%) | 65 (79.3%) |  |
|  |  | Stage IV | 2 (1.4%) | 2 (3.1%) | 0 (0%) |  |
|  | **Maximum probing depth 4 mm** | Stage I | 91 (62.3%) | 39 (60.9%) | 52 (63.4%) | 0.810 † |
|  |  | Stage II | 45 (30.8%) | 21 (32.8%) | 24 (29.3%) |  |
|  |  | Stage III | 9 (6.2%) | 4 (6.3%) | 5 (6.1%) |  |
|  |  | Stage IV | 1 (0.7%) | 0 (0%) | 1 (1.2%) |  |
| **Periodontitis Grading Based on Signs and Symptoms** | **No bone loss over 5 years, smokes 5 cigarettes/day** | Grade A | 26 (17.8%) | 12 (18.8%) | 14 (17.1%) | 0.908 † |
|  |  | Grade B | 117 (80.1%) | 51 (79.7%) | 66 (80.5%) |  |
|  |  | Grade C | 3 (2.1%) | 1 (1.6%) | 2 (2.4%) |  |
|  | **40% bone loss in 30-year-old** | Grade A | 2 (1.4%) | 2 (3.1%) | 0 (0%) | 0.007 †* |
|  |  | Grade B | 39 (26.7%) | 24 (37.5%) | 15 (18.3%) |  |
|  |  | Grade C | 105 (71.9%) | 38 (59.4%) | 67 (81.7%) |  |
|  | **10% bone loss in 50 years old** | Grade A | 108 (74.0%) | 45 (70.3%) | 63 (76.8%) | 0.133 † |
|  |  | Grade B | 27 (18.5%) | 11 (17.2%) | 16 (19.5%) |  |
|  |  | Grade C | 11 (7.5%) | 8 (12.5%) | 3 (3.7%) |  |
|  | **15% bone loss in a diabetic patient with HbA1c of 8** | Grade A | 2 (1.4%) | 1 (1.6%) | 1 (1.2%) | 0.190 † |
|  |  | Grade B | 21 (14.4%) | 13 (20.3%) | 8 (9.8%) |  |
|  |  | Grade C | 123 (84.2%) | 50 (78.1%) | 73 (89.0%) |  |
| **Total Knowledge Score** | **Total correct answers of knowledge questions out of 16** | **Mean ± SD** | 12.6 ± 2.5 | 11.9 ± 2.3 | 13.1 ± 2.5 | 0.005 ‡* |

The responses in this table represent different answer choices from the questionnaire assessing students' ability to diagnose, stage, and grade periodontal diseases according to diagnostic criteria, with the correct answers underlined. Chi-square (†) and independent sample t-tests (‡) were used to assess differences between groups. Statistically significant differences are denoted by *p* < 0.05 (*).

**Supplemental Table 3. Clinical Cases Diagnosis Correctness**

| **Case Number** | **Question** | **Response** | **Total**  **(N=146)** | **5th Year (n=64)** | **6th Year (n=82)** | **p-value** |
| --- | --- | --- | --- | --- | --- | --- |
| **Case 1** | **What is your periodontal diagnosis?** | Gingival health | 3 (2.1%) | 2 (3.1%) | 1 (1.2%) | 0.710 † |
|  |  | Gingivitis | 2 (1.4%) | 1 (1.6%) | 1 (1.2%) |  |
|  |  | Periodontitis | 141 (96.6%) | 61 (95.3%) | 80 (97.6%) |  |
|  | **What is the stage?** | Stage I | 0 (0%) | 0 (0%) | 0 (0%) | 0.182 † |
|  |  | Stage II | 2 (1.4%) | 2 (3.1%) | 0 (0%) |  |
|  |  | Stage III | 118 (80.8%) | 54 (84.4%) | 64 (78.0%) |  |
|  |  | Stage IV | 24 (16.4%) | 7 (10.9%) | 17 (20.7%) |  |
|  |  | Not periodontitis case | 2 (1.4) | 1 (1.6) | 1 (1.2) |  |
|  | **What is the grade?** | Grade A | 35 (24.0%) | 20 (31.3%) | 15 (18.3%) | 0.094 † |
|  |  | Grade B | 75 (51.4%) | 34 (53.1%) | 41 (50.0%) |  |
|  |  | Grade C | 33 (22.6%) | 9 (14.1%) | 24 (29.3%) |  |
|  |  | Not periodontitis case | 33 (2.6) | 9 (14.1) | 24 (29.3) |  |
|  | **What is your justification?** | Bleeding on probing | 5 (3.4%) | 1 (1.6%) | 4 (4.9%) | 0.340 † |
|  |  | Plaque index | 2 (1.4%) | 1 (1.6%) | 1 (1.2%) |  |
|  |  | Probing depth | 4 (2.7%) | 4 (6.3%) | 0 (0%) |  |
|  |  | Attachment loss | 27 (18.5%) | 13 (20.3%) | 14 (17.1%) |  |
|  |  | Bone loss percentage | 61 (41.8%) | 25 (39.1%) | 36 (43.9%) |  |
|  |  | Case complexity | 45 (30.8%) | 19 (29.7%) | 26 (31.7%) |  |
|  |  | Tooth loss due to periodontitis | 2 (1.4) | 1 (1.6) | 1 (1.2) |  |
|  |  | others | 0 | 0 | 0 |  |
| **Case 2** | **What is your periodontal diagnosis?** | Gingival health | 6 (4.1%) | 3 (4.7%) | 3 (3.7%) | 0.798 † |
|  |  | Gingivitis | 38 (26.0%) | 15 (23.4%) | 23 (28.0%) |  |
|  |  | Periodontitis | 102 (69.9%) | 46 (71.9%) | 56 (68.3%) |  |
|  | **What is the stage?** | Stage I | 0 (0%) | 0 (0%) | 0 (0%) | 0.450 † |
|  |  | Stage II | 14 (9.6%) | 9 (14.1%) | 5 (6.1%) |  |
|  |  | Stage III | 86 (58.9%) | 36 (56.3%) | 50 (61.0%) |  |
|  |  | Stage IV | 10 (6.8%) | 4 (6.3%) | 6 (7.3%) |  |
|  |  | Not a periodontitis case | 36 (24.7%) | 15 (23.4%) | 21 (25.6%) |  |
|  | **What is the grade?** | Grade A | 30 (20.5%) | 17 (26.6%) | 13 (15.9%) | 0.417 † |
|  |  | Grade B | 30 (20.5%) | 13 (20.3%) | 17 (20.7%) |  |
|  |  | Grade C | 53 (36.3%) | 20 (31.3%) | 33 (40.2%) |  |
|  |  | Not a periodontitis case | 33 (22.6%) | 14 (21.9%) | 19 (23.2%) |  |
|  | **What is your justification?** | Bleeding on probing | 33 (22.6%) | 11 (17.2%) | 22 (26.8%) | 0.015 †* |
|  |  | Plaque index | 5 (3.4%) | 5 (7.8%) | 0 (0%) |  |
|  |  | Probing depth | 6 (4.1%) | 4 (6.3%) | 2 (2.4%) |  |
|  |  | Attachment loss | 27 (18.5%) | 8 (12.5%) | 19 (23.2%) |  |
|  |  | Bone loss percentage | 65 (44.5%) | 34 (53.1%) | 31 (37.8%) |  |
|  |  | Case complexity | 9 (6.2%) | 2 (3.1%) | 7 (8.5%) |  |
|  |  | Tooth loss due to periodontitis | 0 | 0 | 0 |  |
|  |  | others | 0 | 0 | 0 |  |
| **Case 3** | **What is your periodontal diagnosis?** | Gingival health | 2 (1.4%) | 2 (3.1%) | 0 (0%) | 0.086 † |
|  |  | Gingivitis | 3 (2.1%) | 0 (0%) | 3 (3.7%) |  |
|  |  | Periodontitis | 141 (96.6%) | 62 (96.9%) | 79 (96.3%) |  |
|  | **What is the stage?** | Stage I | 0 (0%) | 0 (0%) | 0 (0%) | 0.677 † |
|  |  | Stage II | 1 (0.7%) | 1 (1.6%) | 0 (0%) |  |
|  |  | Stage III | 63 (43.2%) | 26 (40.6%) | 37 (45.1%) |  |
|  |  | Stage IV | 80 (54.8%) | 36 (56.3%) | 44 (53.7%) |  |
|  |  | Not a periodontitis case | 2 (1.4%) | 1 (1.2%) | 1 (1.2%) |  |
|  | **What is the grade?** | Grade A | 3 (2.1%) | 1 (1.6%) | 2 (2.4%) | 0.696 † |
|  |  | Grade B | 19 (13.0%) | 8 (12.5%) | 11 (13.4%) |  |
|  |  | Grade C | 123 (84.2%) | 54 (84.4%) | 69 (84.1%) |  |
|  |  | Not a periodontitis case | 1 (0.7%) | 1 (1.6%) | 0 |  |
|  | **What is your justification?** | Bleeding on probing | 2 (1.4%) | 1 (1.6%) | 1 (1.2%) | 0.662 † |
|  |  | Plaque index | 2 (1.4%) | 1 (1.6%) | 1 (1.2%) |  |
|  |  | Probing depth | 2 (1.4%) | 1 (1.6%) | 1 (1.2%) |  |
|  |  | Attachment loss | 22 (15.1%) | 10 (15.6%) | 12 (14.6%) |  |
|  |  | Bone loss percentage | 60 (41.1%) | 31 (48.4%) | 29 (35.4%) |  |
|  |  | Case complexity | 41 (28.1%) | 13 (20.3%) | 28 (34.1%) |  |
|  |  | Tooth loss due to periodontitis | 17 (11.3) | 7 (10.9) | 10 (12.2) |  |
|  |  | others | 0 | 0 | 0 |  |
| **Case 4** | **What is your periodontal diagnosis?** | Gingival health | 3 (2.1%) | 2 (3.1%) | 1 (1.2%) | 0.638 † |
|  |  | Gingivitis | 68 (46.6%) | 28 (43.8%) | 40 (48.8%) |  |
|  |  | Periodontitis | 75 (51.4%) | 34 (53.1%) | 41 (50.0%) |  |
|  | **What is the stage?** | Stage I | 0 (0%) | 0 (0%) | 0 (0%) | 0.552 † |
|  |  | Stage II | 25 (17.1%) | 9 (14.1%) | 16 (19.5%) |  |
|  |  | Stage III | 48 (32.9%) | 24 (37.5%) | 24 (29.3%) |  |
|  |  | Stage IV | 7 (4.8%) | 4 (6.3%) | 3 (3.7%) |  |
|  |  | Not a periodontitis case | 66 (45.2%) | 27 (42.2%) | 39 (47.6%) |  |
|  | **What is the grade?** | Grade A | 30 (20.5%) | 12 (18.8%) | 18 (22.0%) | 0.353 † |
|  |  | Grade B | 29 (19.9%) | 17 (26.6%) | 12 (14.6%) |  |
|  |  | Grade C | 21 (14.4%) | 8 (12.5%) | 13 (15.9%) |  |
|  |  | Not a periodontitis case | 66 (45.2%) | 27 (42.2%) | 39 (47.6%) |  |
|  | **What is your justification?** | Bleeding on probing | 53 (36.3%) | 19 (29.7%) | 34 (41.5%) | 0.220 † |
|  |  | Plaque index | 11 (7.5%) | 6 (9.4%) | 5 (6.1%) |  |
|  |  | Probing depth | 14 (9.6%) | 8 (12.5%) | 6 (7.3%) |  |
|  |  | Attachment loss | 22 (15.1%) | 8 (12.5%) | 14 (17.1%) |  |
|  |  | Bone loss percentage | 42 (28.8%) | 22 (34.4%) | 20 (24.4%) |  |
|  |  | Case complexity | 3 (2.1%) | 0 (0%) | 3 (3.7%) |  |
|  |  | Tooth loss due to periodontitis | 0 | 0 | 0 |  |
|  |  | others | 1 (0.7) | 1 (1.6) | 0 |  |
| **Case 5** | **What is your periodontal diagnosis?** | Gingival health | 7 (4.8%) | 5 (7.8%) | 2 (2.4%) | 0.117 † |
|  |  | Gingivitis | 105 (71.6%) | 41 (64.1%) | 64 (78.0%) |  |
|  |  | Periodontitis | 34 (23.3%) | 18 (28.1%) | 16 (19.5%) |  |
|  | **What is the stage?** | Stage I | 0 (0%) | 0 (0%) | 0 (0%) | 0.494 † |
|  |  | Stage II | 8 (5.5%) | 5 (7.8%) | 3 (3.7%) |  |
|  |  | Stage III | 27 (18.5%) | 14 (21.9%) | 13 (15.9%) |  |
|  |  | Stage IV | 2 (1.4%) | 1 (1.6%) | 1 (1.2%) |  |
|  |  | Not a periodontitis case | 109 (74.7%) | 44 (68.8%) | 65 (79.3%) |  |
|  | **What is the grade?** | Grade A | 15 (10.3%) | 8 (12.5%) | 7 (8.5%) | 0.195 † |
|  |  | Grade B | 15 (10.3%) | 10 (15.6%) | 5 (6.1%) |  |
|  |  | Grade C | 9 (6.2%) | 3 (4.7%) | 6 (7.3%) |  |
|  |  | not a periodontitis case | 107 (73.3%) | 43 (67.2%) | 64 (78.0%) |  |
|  | **What is your justification?** | Bleeding on probing | 84 (57.5%) | 35 (54.7%) | 49 (59.8%) | 0.240 † |
|  |  | Plaque index | 11 (7.5%) | 4 (6.3%) | 7 (8.5%) |  |
|  |  | Probing depth | 10 (6.8%) | 7 (10.9%) | 3 (3.7%) |  |
|  |  | Attachment loss | 19 (13.0%) | 8 (12.5%) | 11 (13.4%) |  |
|  |  | Bone loss percentage | 9 (6.2%) | 6 (9.4%) | 3 (3.7%) |  |
|  |  | Case complexity | 5 (3.4%) | 3 (4.7%) | 2 (2.4%) |  |
|  |  | Tooth loss due to periodontitis | 2 (1.4%) | 1 (1.6%) | 1 (1.2%) |  |
|  |  | Others | 5 (3.4%) | 0 (0%) | 5 (6.1%) |  |
| **Total diagnostic accuracy score out of 15 (mean ± SD)** | | | 9.3 ± 2.4 | 9.1 ± 2.6 | 9.5 ±2.2 | 0.313 ‡ |

The responses in this table represent students' diagnostic accuracy in periodontal diagnosis, staging, and grading for five clinical cases, with the correct answers underlined. Chi-square (†) and independent sample t-tests (‡) were used to assess differences between groups. Statistically significant differences are denoted by *P* < 0.05 (*).
